# Pernicious Anæmia

**Published:** 1906-03-17

**Authors:** 


### PERNICIOUS ANÆMIA.

In a lecture on this disease Dr. Hale White [1] gave considerable excerpts from the original account of the disease given fifty years ago by its discoverer, Addison, of Guy's; and since the text is not now readily accessible, it is worth while to reproduce a description so good that little remains to be added to it even after so long a lapse of time. The account appears incidentally in a book entitled "On the Constitutional and Local Effects of Disease of the Supra-renal Capsule," the book, that is to say, descriptive of the disease which still bears Addison's name.

"For a long period," he says, "I have from time to time met with a very remarkable form of general anæmia, occurring without any discoverable cause whatever, cases in which there had been no previous loss of blood, no existing diarrhœa, no chlorosis, no purpura, no renal or splenic, miasmic, glandular, strumous, or malignant disease. Accordingly, in speaking of this form of anæmia in a clinical lecture, I propose, with little propriety, to apply the term 'idiopathic' to distinguish it from cases in which there occurs more or less evidence of some of the usual causes or concomitants of the anæmic state. The disease presented in every instance the same general character, pursued a similar course, and, with scarcely a single accident, was followed, after a period, by the same fatal result. It occurs in both sexes, generally, not exclusively, beyond the middle period of life, and so far as I know at present, chiefly in persons of a somewhat large and bulky frame, and with a strongly marked tendency to the formation of fat. It makes its approach with so slow and insidious a manner that the patient can hardly fix a date to his earliest feelings of that langour which is shortly to become so extreme. The countenance gets pale, the whites of the eyes become pearly, the general frame flabby rather than wasted, the pulse perhaps large but remarkably soft and compressible, and occasionally with a slight jerk, especially under the slightest exertion. There is an increasing indisposition to exertion, with an uncomfortable feeling of faintness or breathlessness on attempting it. The heart is readily made to palpitate. The whole surface of the body presents a blanched, smooth, waxy appearance. Extreme langour and faintness supervene, breathlessness and palpitations being produced by the most trifling exertion or emotion. Some trifling œdema is probably perceived about the eyelids, debility becomes extreme, the patient can no longer rise from his bed, the mind occasionally wanders, he falls into a prostrate and half-torpid state, and at length expires. Nevertheless, to the very last, after a sickness of several months' duration, the bulkiness of the general frame of the man often presents a most striking contrast to the failure and exhaustion observed in every other respect."

[1] Clin. Jour., Feb. 28, 1906.

